# Pregnancy and its outcomes in hemodialysis patients in Turkey

**DOI:** 10.3906/sag-2106-120

**Published:** 2021-11-27

**Authors:** Hamad DHEİR, Özkan GÜNGÖR, Sena ULU, Ebru GÖK OĞUZ, Necmi EREN, Orçun ALTUNÖREN, Erhan TATAR, Gökhan ATILGAN, Süleyman KARAKÖSE, İbrahim GÜNEY, Eray EROĞLU, Rüya MUTLUAY, İlter BOZACI, Alper ALP, Hakan AKDAM, Meltem Seziş DEMİRCİ, Zeki SOYPAÇACI, Özger AKARSU, Saime PAYDAŞ, Zafer ERCAN, Ekrem KARA, Cevat TOPAL, Hakan YAVAŞ, Nihan TEKKARIŞMAZ, Kenan TURGUTALP, Can HÜZMELİ, Ayça İNCİ, Güner KARAVELİ GÜRSOY, Ayşe Jini GÜNEŞ KESKİN, Bülent HUDDAM, Ender HÜR, Abdülmecit YILDIZ, Garip BEKFİLAVİOĞLU, Tuncay ŞAHUTOĞLU, Mehmet TUNCAY, Simge BARDAK, Serkan BAKIRDOĞEN, Zülfikar YILMAZ, Emrah GÜNAY, Onur TUNCA, Sinan KAZAN, İrem Pembegül YİĞİT, Hazen SARITAŞ, Can SEVİNÇ, Hakan KAPTANOĞULLARI, Sibel GÖKÇAY BEK, İlhan KURULTAK, Ali DEĞİRMENCİ, Murat ŞAKACI, İlhan KILIÇ, Zeki AYDIN, Hülya ÇOLAK, Erkan DERVİŞOĞLU, Garip ŞAHİN, Mehmet Deniz AYLI, Savaş SİPAHİ, Ülver DERİCİ

**Affiliations:** 1 Division of Nephrology, Department of Internal Medicine, Faculty of Medicine, Sakarya University, Sakarya, Turkey; 2 Division of Nephrology, Department of Internal Medicine, Faculty of Medicine, Kahramanmaraş Sütçü İmam University, Kahramanmaraş, Turkey; 3 Division of Nephrology, Department of Internal Medicine, Faculty of Medicine, Başakşehir University, İstanbul, Turkey; 4 Division of Nephrology, Department of Internal Medicine, Ankara Dıskapı Training and Research Hospital, University of Health Sciences, Ankara, Turkey; 5 Division of Nephrology, Department of Internal Medicine, Faculty of Medicine, Kocaeli University, Kocaeli, Turkey; 6 Division of Nephrology, Department of Internal Medicine, İzmir Bozyaka Training and Research Hospital, University of Health Sciences, İzmir, Turkey; 7 Division of Nephrology, Department of Internal Medicine, Konya Training and Research Hospital, University of Health Sciences, Konya, Turkey; 8 Division of Nephrology, Department of Internal Medicine, Faculty of Medicine, Erciyes University, Kayseri, Turkey; 9 Division of Nephrology, Department of Internal Medicine, Faculty of Medicine, Osmangazi University, Eskişehir, Turkey; 10 Division of Nephrology, Department of Internal Medicine, Faculty of Medicine, Muğla Sıtkı Kocaman University, Muğla, Turkey; 11 Division of Nephrology, Department of Internal Medicine, Faculty of Medicine, Adnan Menderes University, Aydın, Turkey; 12 Division of Nephrology, Department of Internal Medicine, Faculty of Medicine, Ege University, İzmir, Turkey; 13 Division of Nephrology, Department of Internal Medicine, Katip Çelebi Training and Research Hospital, İzmir, Turkey; 14 Division of Nephrology, Department of Internal Medicine, Bursa Training and Research Hospital, University of Health Sciences, Bursa, Turkey; 15 Division of Nephrology, Department of Internal Medicine, Faculty of Medicine, Çukurova University, Adana, Turkey; 16 Division of Nephrology, Department of Internal Medicine, Bartın State Hospital, Bartın, Turkey; 17 Division of Nephrology, Department of Internal Medicine, Faculty of Medicine, Recep Tayyip Erdoğan University, Rize, Turkey; 18 Division of Nephrology, Department of Internal Medicine, Trabzon Medicalpark Hospital Complex, Trabzon, Turkey; 19 Division of Nephrology, Department of Internal Medicine, Tokat State Hospital, Tokat, Turkey; 20 Division of Nephrology, Department of Internal Medicine, Adana Application and Research Center, Başkent University, Adana, Turkey; 21 Division of Nephrology, Department of Internal Medicine, Faculty of Medicine, Mersin University, Mersin, Turkey; 22 Division of Nephrology, Department of Internal Medicine, Hatay State Hospital, Hatay, Turkey; 23 Division of Nephrology, Department of Internal Medicine, Antalya Training and Research Hospital, Antalya, Turkey; 24 Division of Nephrology, Department of Internal Medicine, Osmaniye State Hospital, Osmaniye, Turkey; 25 Division of Nephrology, Department of Internal Medicine, Mersin State Hospital, Mersin, Turkey; 26 Division of Nephrology, Department of Internal Medicine, Manisa Merkezefendi State Hospital, Manisa, Turkey; 27 Division of Nephrology, Department of Internal Medicine, Faculty of Medicine, Uludağ University, Bursa, Turkey; 28 Division of Nephrology, Department of Internal Medicine, Adıyaman Training and Research Hospital, Adıyaman, Turkey; 29 Division of Nephrology, Department of Internal Medicine, Mehmet Akif İnan Training and Research Hospital, Şanlıurfa, Turkey; 30 Division of Nephrology, Department of Internal Medicine, Dr Ersin Arslan Training and Research Hospital, Gaziantep, Turkey; 31 Division of Nephrology, Department of Internal Medicine, Dr. Burhan Nalbantoğlu State Hospital, Turkish Republic of north Cyprus; 32 Division of Nephrology, Department of Internal Medicine, Faculty of Medicine, Çanakkale Onsekiz Mayıs University, Çanakkale, Turkey; 33 Division of Nephrology, Department of Internal Medicine, Faculty of Medicine, Dicle University, Diyarbakır, Turkey; 34 Division of Nephrology, Department of Internal Medicine, Gaziyaşargil Training and Research Hospital, Diyarbakır, Turkey; 35 Division of Nephrology, Department of Internal Medicine, Mardin State Hospital, Mardin, Turkey; 36 Division of Nephrology, Department of Internal Medicine, Faculty of Medicine, Afyon Kocatepe University, Afyon, Turkey; 37 Division of Nephrology, Department of Internal Medicine, Faculty of Medicine, Malatya Turgut Özal University, Malatya, Turkey; 38 Division of Nephrology, Department of Internal Medicine, Siirt State Hospital, Siirt, Turkey; 39 Division of Nephrology, Department of Internal Medicine, Erzurum Training and Research Hospital, Erzurum, Turkey; 40 Division of Nephrology, Department of Internal Medicine, Faculty of Medicine, Biruni University Vocational School of Health, İstanbul, Turkey; 41 Division of Nephrology, Department of Internal Medicine, Faculty of Medicine, Trakya University, Edirne, Turkey; 42 Division of Nephrology, Department of Internal Medicine, Çanakkale State Hospital, Çanakkale, Turkey; 43 Division of Nephrology, Department of Internal Medicine, Tekirdağ State Hospital, Tekirdağ, Turkey; 44 Division of Nephrology, Department of Internal Medicine, Kırklareli State Hospital, Kırklareli, Turkey; 45 Division of Nephrology, Department of Internal Medicine, Darıca Farabi Training and Research Hospital, Kocaeli, Turkey; 46 Division of Nephrology, Department of Internal Medicine, Tepecik Training and Research Hospital, University of Health Sciences, İzmir, Turkey; 47 Division of Nephrology, Department of Internal Medicine, Faculty of Medicine, Gazi University, Ankara, Turkey

**Keywords:** Chronic renal failure, hemodialysis, pregnancy, dialysis session, fatal outcome, infertility

## Abstract

**Background/aim:**

This study aimed to investigate pregnancy frequency and evaluate the factors affecting live births in hemodialysis (HD) patients.

**Materials and methods:**

Female HD patients whose pregnancy was retrospectively reported between January 1, 2014, and December 31, 2019. The duration of HD, primary disease, and the information on whether the pregnancy resulted in abortion, stillbirth, or live birth, whether the HD duration was prolonged after diagnosing the pregnancy and whether it accompanied preeclampsia were recorded.

**Results:**

In this study, we reached 9038 HD female patients’ data in the study. A total of 235 pregnancies were detected in 145 patients. The mean age was 35.42 (35 ± 7.4) years. The mean age at first gestation was 30.8 ± 6.5 years. The average birth week was 32 (28–36) weeks. A total of 53.8% (no = 78) of the patients had live birth, 51.7% (no = 70) had at least one abortion in the first 20 weeks, and 13.1% (no = 19) had at least one stillbirth after 20 weeks. The rate of patients’ increased numbers of dialysis sessions during pregnancy was 71.7%. The abortion rate was 22.4% in those with increased HD sessions, whereas 79.3% in those not increased HD sessions (p < 0.001). Live birth frequency was 67.2% in the increased HD sessions group and 3.4% in those who did not differ in HD sessions (p < 0.001).

**Conclusion:**

For the first time, we reported pregnancy outcomes in HD female patients, covering all regions of Turkey. It has been observed that; increasing the number of HD sessions in dialysis patients will decrease fetal and maternal complications and increase live birth rates.

## 1. Introduction

End-stage kidney disease is associated with low fertility, and women on dialysis are estimated to have a 1/100 chance of becoming pregnant compared to the general population [1,2]. Many abnormalities include low follicle-stimulating hormone (FSH), luteinizing hormone (LH), progesterone, estrogen deficiency, hyperprolactinemia, ovulation inhibition, subclinical hypothyroidism, anemia, mood disorders, and decreased libido are common in uremic patients [3–5]. In addition, endometrial atrophy due to changes in the hypothalamic-pituitary-gonadal axis is common in predialyitic and hemodialysis female patients cause a disrupted ovulation process. Even if the menstrual cycle is regular, implantation impairment may occur due to changes in the pulsatility of hypothalamic-pituitary-gonadal hormones [6,7].

In 1970, pregnancy in a hemodialysis (HD) patient resulting in successful delivery was reported for the first time [8]. In the following years, case reports about pregnancy in HD patients were started to be presented in the literature [9,10]. The information about the frequency of pregnancy in female HD patients is heterogeneous. The reported frequency of pregnancy in women of childbearing age, who are undergoing HD, has increased from 0.54% to 3.3% / 1000 patient-years [11]. The pregnant hemodialysis patient may encounter many complications such as hypertension, miscarriage, premature birth, delivery of a baby with low weight, fetal growth restriction, and fetal and maternal death during pregnancy. Continuously developing HD technology, treating anemia, preserving residual renal functions, and increasing weekly dialysis hours caused increased pregnancy rates and live birth results [11–12]. Increasing the number of conventional weekly HD sessions or extended dialysis, such as nocturnal HD, has been shown to increase live birth rates and, at the same time, reduce the risks of developing the complications mentioned above [13 – 15]. It is challenging to identify and treat the situations such as managing pregnant patients with CKD, determining the optimal treatment method, evaluating the expectant mother before pregnancy, and monitoring the possible complications through pregnancy. The main reason for this is the absence of organized large studies. This study aims to investigate the pregnancy frequency and outcomes in female HD patients in Turkey.

## 2. Materials and methods

This study analyzed the information of HD female patients whose pregnancy status was reported between January 1, 2014 and December 31, 2019 across Turkey. This study was approved by Sakarya University Ethics Committee (No: 71522473/050.01.04/295). We divided the regions of the country into seven parts and designated accountable nephrologists for each area. A total of 9038 female patient data were obtained by contacting nephrologists from other provinces through accountable nephrologists.

All study patients who 1) are over 18 years old, 2) have a history of pregnancy, and 3) reached to other pregnancy information were included. Patients undergoing nocturnal HD or peritoneal dialysis were excluded from the study. The patients’ age, HD duration, primary disease, the information on whether the pregnancy resulted in abortion, stillbirth, or live birth or not if HD period was prolonged after learning the pregnancy, and if preeclampsia was accompanied or not were recorded.

### 2.1. Statistical analysis

SPSS (Statistical Package for Social Sciences) version 22.0 program was used for statistical analysis in evaluating the data. Descriptive statistical data were shown as frequency (percentage), median (minimum-maximum) (25th percentile–75th percentile), and mean ± standard deviation. Distribution characteristics of numerical variables were evaluated by using the Kolmogorov–Smirnov test. The chi-square test was used in the comparison of categorical data. The Mann–Whitney U test was used to compare the variables that were not normally distributed. Categorical features and relationships between groups were assessed using an appropriate chi-square test. The p value <0.05 was accepted as statistically significant.

## 3. Results

A total of 9038 female patients were included in the study. A total of 235 pregnancy histories were detected in 145 patients. Some of the 145 patients had more than one pregnancy history, and we found 235 pregnancy histories at the end of the study. The mean age of the patients was 35.42 (35 ± 7.4) years. The most common primary diseases of the patients were diabetes mellitus (17%), hypertension (9.7%), glomerulonephritis (17%), and polycystic kidney disease (12.8%). The mean HD duration was 72 (36–139) months. The mean first pregnancy age was 30.8 ± 6.5 years. 53.8% (no = 78) of the patients had live birth, 51.7% (no = 70) had at least one abortion in the first 20 weeks, and 13.1% (no = 19) had at least one stillbirth after 20 weeks.

**Figure 1:**
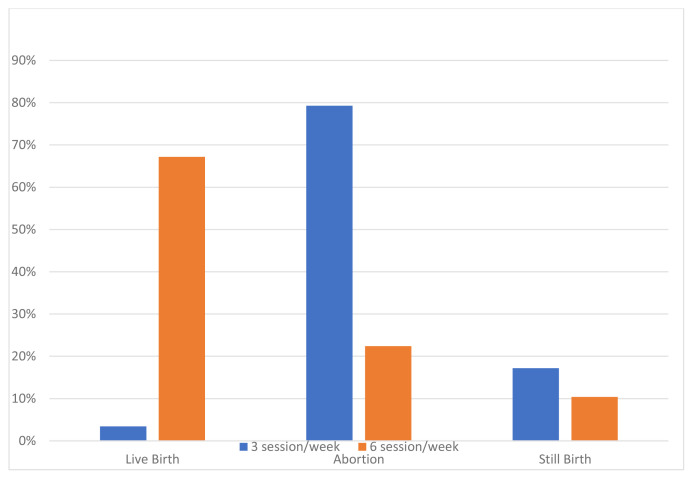
The relation between the number of weekly hemodialysis sessions and the outcomes of pregnancy.

**Figure 2:**
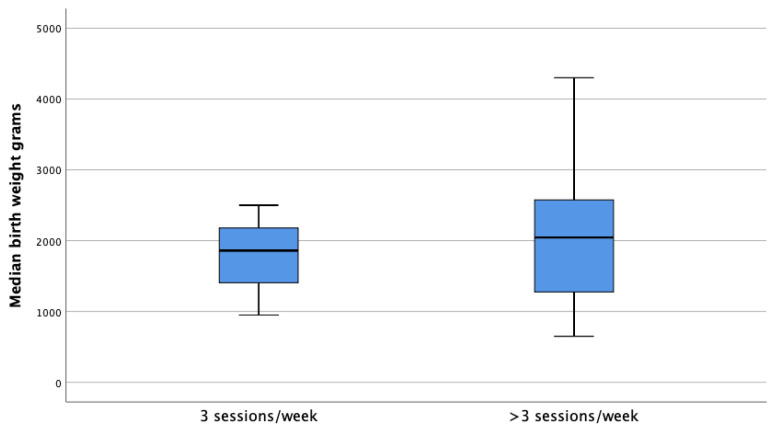
The relationship between birth weight and the number of weekly HD sessions.

The clinical and biological features of the patients are summarized in Table 1. The rate of patients whose dialysis sessions were increased during pregnancy was 71.7%. The average weekly dialysis session was 5 (3–6) sessions. In 73.1% of cases, delivery was carried out by cesarean method. Of the patients with increased HD sessions during pregnancy, 67.2% resulted in a live birth, 22.4% abortion, and 10.4% stillbirth. Besides, of those whose HD sessions were not increased, 3.4% resulted in live birth, 79.3% in abortion, and 17.2% in stillbirth (p < 0.001) (Table 2). Figure 1 shows the relationship between increased weekly HD sessions and successful pregnancy processes. The mean live birth week was 32 (28 – 36 weeks) weeks. The mean newborn birth weight was 1966.03 ± 816.17 grams. In terms of median birth weight, in patients who resulted in a live birth it was 1860 (950–2500) g in the group whose HD sessions were not increased, whereas, in the group with increased weekly HD sessions, it was 2045 (1275–2575) g higher (Figure 2). This result was not statistically significant (p = 0.678). Preeclampsia was reported in 24 (10.2%) cases.

## 4. Discussion

In his study, for the first time, pregnancy rates, dialysis application profile during pregnancy, and pregnancy outcomes were revealed by retrospectively screening a large group of female HD patients, which is the largest epidemiological study covering all regions of Turkey.

Despite the improvements of dialysis methods, their effectiveness, and the membranes, pregnancy incidence in uremic patients is still very low. The incidence of pregnancy varies between 1% and 7% in HD patients. [16,17]. Although HD patients maintain their pregnancies, maternal and fetal complications are common. First pregnancy reports of successful live birth rates in HD patients were extremely low [18]. These rates increased up to 50% in the 2000s because of the advancement of dialysis efficacy [19]. Reported data on pregnancy outcomes and management in uremic female patients varies from country to country in the world [20]. Until now, there was no clear data on this issue in our country. With this study, pregnancy outcomes were studied in conventional HD patients for the first time in Turkey, with 57 nephrologists covering seven geographical regions. More than half of the patients’ pregnancies (53.8%) resulted in a live birth, while the remaining half resulted in abortion or stillbirth. The mean weights of alive babies were 1966.03 ± 816.17 grams. In a similar study by Malik et al., live birth rates were 58%, and the average of babies’ birth weights were 1700 grams [17].

Premature birth rates are quite high in HD patients. Moreover, premature birth is the most important cause of death in newborn babies. Our study determined the mean birth week of the patients as 32 (28–36) weeks. In a study of 28 HD patients, 18 patients (64.2%) had a mean week of successful live birth of 32 weeks and a mean birth weight of 1747.4 ± 607.0 g [21]. Similarly, Eroglu et al., in a small-scale study, showed that the mean gestational age at delivery was 32 weeks, and the mean newborn birthweight was 1400 (420–2640 grams) g in 7 HD pregnant patients [22].

The fundamental approach to achieving successful pregnancy results is to increase the weekly dialysis dose. [12,23]. It is possible to reduce premature birth rates, achieve high birth weights, and deliver at term by performing intensive or prolonged HD [12, 24–26]. The literature data show that the incidence of pregnancy has increased by raising the number of dialysis sessions in the last two decades [11]. In a study conducted by Sachdeva et al., 78% of 187 pregnant women had a live birth. In 61% of patients, dialysis sessions were increased to 6 sessions/week (mean 5.5 ± 1.1 sessions) [27]. Similarly, in our study, the average number of weekly dialysis was 5 (3–6) days, and the rate of patients whose dialysis sessions increased during pregnancy was 71.7%. In this retrospective study, we did not find information about why the number of HD sessions was not increased in some pregnant patients from the recorded files. The possible reasons for this may be that these patients could not reach the nephrologist in the area where they live, lack of accountable nephrologists in some dialysis centers, and some patients may be having 1–2 sessions or up to three sessions per week. Nearly two-thirds (67.2%) of those with increased HD sessions during pregnancy resulted in live births and one-third with abortion or stillbirth. A total of 96% of patients whose HD sessions were not increased resulted in abortion or stillbirth, whereas only 3% resulted in a live birth. These results indicate the increased possibility of acquiring positive results by increasing the dialysis dose during pregnancy via reducing exposure to uremia. Although weekly dialysis hours are recommended as > 20 h/week, in a study recently published by the Toronto group, the live birth rate has been shown to increase to around 85% by nocturnal dialysis with at least 36 h a week [12]. Also, compared to conventional dialysis, longer gestational weeks and, thus, higher birth weight and birth rates were obtained with nocturnal dialysis.

The most important cause of abortion and premature births in uremic pregnant patients is preeclampsia. A recently published study showed that preeclampsia developed in 15 of 40 pregnant patients (37.5%) with diagnosed stage 4 – 5 CKD. Of those, ten patients had an abortion, and 29 patients had a premature birth. Only one patient had a timely delivery. It has been reported that 5 of the prematurely born babies died [28]. In another study investigating the factors affecting fetal outcomes in 93 pregnant HD patients, preeclampsia rates were found around 15%. It has been shown that detected preeclampsia shortens the gestational week, negatively affects successful live birth rates and is responsible for 40% of perinatal deaths. Besides, 53% are associated with various adverse outcomes. In addition, all babies born alive from preeclamptic patients were premature, and 9 of them were found to be advanced prematurely [29]. On the other hand, it has been shown that the risk of developing preeclampsia significantly decreases as the weekly dialysis sessions increase [30]. Our study found that 10.2% of the patients who completed 20 weeks of gestation developed preeclampsia. We evaluated this rate lower than the literature data. We consider that; retrospective data, close monitoring of the nephrologists, and prolonging the weekly dialysis session may have affected these rates.

The limitations of the present study are as follows: a retrospective nature, the inability to obtain information about whether stillborn babies have chromosomal abnormalities, unavailability of records of hemoglobin values at the beginning and throughout the pregnancy to compare live births and stillbirths outcomes, and the inability to obtain sufficient data on anemia management such as erythropoietin therapy.

In conclusion, live birth rates in HD patients are higher than the ones in the ancient times due to HD efficacy, development of membranes, increased weekly dialysis sessions, obstetrics, and neonatal care. In order to maintain a successful pregnancy process, it should be aimed to reduce the exposure of dialysis patients to uremia during the week. Increasing the number of weekly HD sessions is essential in this respect. Controlling the weekly dry weight, ensuring adequate daily maternal-fetal calories, management of comorbid conditions such as hypertension and anemia are imperative. We believe that adopting these approaches will reduce fetal and maternal complications and, thus, increase successful live birth rates.

## Figures and Tables

**Table 1:** Demographic and clinical characteristics of patients.

Characteristics	Outcome
Age (years)	35.42 (35 ± 7.4)
Hemodialysis duration, means, (Months)	72 (36–139)
**Primary Disease**	
Diabetes mellitus	17 (11.7%)
Hypertension	14 (9.7%)
Glomerulonephritis	17 (11.7%)
Polycystic kidney disease	4 (12.8%)
Nephrolithiasis	9 (6.2%)
Vesicoureteral reflux	10 (6.9%)
Unknown	22 (15.1%)
Others	52 (35.9%)
Total pregnancy numbers, (no)	235
Mean first pregnancy age, (Years)	30.8 ± 6.5
Gestation age in dialysis, (Months)	19.0 (7.0–40.0)
The rate of increasing dialysis sessions during pregnancy, (%)	71.7
At least one abortion in the first 20 weeks, no, (%)	70 (51.7)
At least one stillbirth after 20 weeks, no, (%)	19 (13.1)
Number of live births, no, (%)	78 (53.8)
Cesarean/Vaginal delivery rates, (%)	39.3/14.5
Mean live birth weight, (grams)	1966.03 ± 316.17
Mean number of weekly dialysis sessions, (n)	5.0 (3.0–6.0)
Mean birth week, no, (%)	32.0 (28.0–36.0)
Frequency of preeclampsia, no, (%)	24 (10.2)
Maternal death, (%)	0

**Table 2:** The relation between the increase in the number of HD sessions and the course of pregnancy.

	Live birth (%)	Abortion (%)	Stillbirth (%)	p value
**No increase in the number of HD sessions, (%)**	3.4	79.3	17.2	**< 0.001**
**İncreased number of HD sessions, (%)**	67.2	22.4	10.4	**< 0.001**

## References

[1] Piccoli GB Cabiddu G Daidone G Guzzo G Maxia S The children of dialysis: live-born babies from on-dialysis mothers in Italy--an epidemiological perspective comparing dialysis, kidney transplantation and the overall population Nephrology Dialysis Transplantation 2014 29 8 1578 1586 10.1093/ndt/gfu092 24759612

[2] Hou S Pregnancy in chronic renal insufficiency and end-stage renal disease American Journal of Kidney Diseases 1999 33 2 235 252 10.1016/s0272-6386(99)70296-9 10023634

[3] Dumanski SM Ahmed SB Fertility and reproductive care in chronic kidney disease Journal of nephrology 2019 32 1 39 50 10.1007/s40620-018-00569-9 30604149

[4] Palmer BF Clegg DJ Gonadal dysfunction in chronic kidney disease Reviews in Endocrine and Metabolic Disorders 2017 18 1 117 130 10.1007/s11154-016-9385-9 27586847

[5] Ng HJ Tan WJ Mooppil N Newman S Griva K Prevalence and patterns of depression and anxiety in hemodialysis patients: A 12-month prospective study on incident and prevalent populations British Journal of Health Psychology 2015 20 2 374 395 10.1111/bjhp.12106 24811542

[6] Jones DC Hayslett JP Outcome of Pregnancy in Women with Moderate or Severe Renal Insufficiency New England Journal of Medicine 1996 335 4 226 232 10.1056/NEJM199607253350402 8657238

[7] Katarzyna S Stanisław R Joanna M-R Katarzyna S-S Morphological changes in endometrium of hemodialyzed women of reproductive age Gynecological Endocrinology 2007 23 9 523 526 10.1080/09513590701557523 17943547

[8] Holley JL Reddy SS Pregnancy in dialysis patients: a review of outcomes, complications, and management Seminars in Dialysis 2003 16 5 384 388 10.1046/j.1525-139x.2003.16085.x 12969392

[9] Unzelman RF Alderfer GR Chojnacki RE Pregnancy and chronic hemodialysis Transactions - American Society for Artificial Internal Organs 1973 19 1 144 149 10.1097/00002480-197301900-00026 4722730

[10] Kobayashi H Matsumoto Y Otsubo O Otsubo K Naito T Successful pregnancy in a patient undergoing chronic hemodialysis Obstetrics and Gynecology 1981 57 3 382 386 7465154

[11] Shahir AK Briggs N Katsoulis J Levidiotis V An observational outcomes study from 1966–2008, examining pregnancy and neonatal outcomes from dialysed women using data from the ANZDATA Registry Nephrology (Carlton) 2013 18 4 276 284 10.1111/nep.12044 23441694

[12] Hladunewich MA Hou S Odutayo A Cornelis T Pierratos A Intensive hemodialysis associates with improved pregnancy outcomes: a Canadian and United States cohort comparison Journal of the American Society of Nephrology 2014 25 5 1103 1109 10.1681/ASN.2013080825 24525032 PMC4005313

[13] Giorgina BP Fosca M Elisabetta V Gianfranca C Rossella A Pregnancy in dialysis patients in the new millennium: a systematic review and meta-regression analysis correlating dialysis schedules and pregnancy outcomes Nephrology Dialysis Transplantation 2016 31 11 1915 1934 10.1093/ndt/gfv395 26614270

[14] Michelle AH Susan H Ayodele O Tom C Andreas P Intensive hemodialysis associates with improved pregnancy outcomes: a Canadian and United States cohort comparison Journal of the American Society of Nephrology 2014 25 5 1103 1109 10.1681/ASN.2013080825 24525032 PMC4005313

[15] Moumita B Michelle H Johannes K Andreas P Philip M Successful pregnancies on nocturnal home hemodialysis Clinical Journal of the American Society of Nephrology 2008 3 2 392 396 10.2215/CJN.04110907 18308997 PMC2390936

[16] Hou SH Frequency and outcome of pregnancy in women on dialysis American Journal of Kidney Diseases 1994 23 1 60 63 10.1016/s0272-6386(12)80813-4 8285199

[17] Malik GH Al-Harbi A Al-Mohaya S Dohaimi H Kechrid M Pregnancy in patients on dialysis experience at a referral center Journal of the Association of Physicians of India 2005 53 937 941 16515231

[18] Registration Committee of the European Dialysis and Transplant Association Successful pregnancies in women treated by dialysis and kidney transplantation An International Journal of Obstetrics & Gynaecology 1980 87 10 839 845 10.1111/j.1471-0528.1980.tb04434.x 7000160

[19] Chou C-Y Ting I-W Lin T-H Lee C-N Pregnancy in patients on chronic dialysis: A single center experience and combined analysis of reported results European Journal of Obstetrics & Gynecology and Reproductive Biology 2008 136 2 165 170 10.1016/j.ejogrb.2007.01.017 17560006

[20] Piccoli GB Conijn A Consiglio V Vasario E Attini R Pregnancy in dialysis patients: is the evidence strong enough to lead us to change our counseling policy? Clinical Journal of the American Society of Nephrology 2010 5 1 62 71 10.2215/CJN.05660809 19965547 PMC2801656

[21] Asamiya Y Otsubo S Matsuda Y Kimata N Kikuchi KAN The importance of low blood urea nitrogen levels in pregnant patients undergoing hemodialysis to optimize birth weight and gestational age Kidney International 2009 75 11 1217 1222 10.1038/ki.2009.48 19242506

[22] Eroğlu D Lembet A Ozdemir FN Ergin T Kazanci F Transplantation Proceedings 2004 36 1 53 55 10.1016/j.transproceed.2003.11.002 15013299

[23] Alkhunaizi A Melamed N Hladunewich MA Pregnancy in advanced chronic kidney disease and end-stage renal disease Current Opinion in Nephrology and Hypertension 2015 24 3 252 259 10.1097/MNH.0000000000000119 26066474

[24] Bagon JA Vernaeve H De Muylder X Lafontaine JJ Martens J Pregnancy and dialysis American Journal of Kidney Diseases 1998 31 5 756 765 10.1016/s0272-6386(98)70060-5 9590184

[25] Jungers P Chauveau D Pregnancy in renal disease Kidney International 1997 52 4 871 885 10.1038/ki.1997.408 9328925

[26] Craig KL Podymow T Pauly RP Intensifying renal replacement therapy during pregnancy: the role for nocturnal home hemodialysis International Urology and Nephrology 2010 42 1 137 139 10.1007/s11255-009-9680-4 19937115

[27] Sachdeva M Barta V Thakkar J Sakhiya V Miller I Pregnancy outcomes in women on hemodialysis: a national survey Clinical Kidney Journal 2017 10 2 276 281 10.1093/ckj/sfw130 28396746 PMC5381241

[28] Rivera JCH López MJP Bermúdez CHC Covarrubias LG Aceves LAB Delayed Initiation of Hemodialysis in Pregnant Women with Chronic Kidney Disease: Logistical Problems Impact Clinical Outcomes. An Experience from an Emerging Country Journal of Clinical Medicine 2019 8 4 475 10.3390/jcm8040475 30965626 PMC6518183

[29] Luders C Titan SM Kahhale S Francisco RP Zugaib M Risk Factors for Adverse Fetal Outcome in Hemodialysis Pregnant Women Kidney International Reports 2018 3 5 1077 1088 10.1016/j.ekir.2018.04.013 30197974 PMC6127404

[30] Michelle H Dori S Intensive dialysis and pregnancy Hemodialysis International 2016 20 3 339 348 10.1111/hdi.12420 27061443

